# High Expression of VSTM2L Induced Resistance to Chemoradiotherapy in Rectal Cancer through Downstream IL-4 Signaling

**DOI:** 10.1155/2021/6657012

**Published:** 2021-01-08

**Authors:** Hao Liu, Zhenzhan Zhang, Peilin Zhen, Meijuan Zhou

**Affiliations:** ^1^Jiangmen Central Hospital, Affiliated Jiangmen Hospital of Sun Yat-sen University, Jiangmen, China; ^2^Department of General Surgery, Nanfang Hospital, Southern Medical University, Guangzhou, China; ^3^Department of Radiation Medicine, Guangdong Provincial Key Laboratory of Tropical Disease Research, School of Public Health, Southern Medical University, Guangzhou, China

## Abstract

**Background:**

Preoperative chemoradiotherapy (pCRT) is a common and essential therapeutic strategy for patients with locally advanced rectal cancer (LARC), but poor tumor response and therapeutic resistance to chemoradiotherapy have appeared usually among persons and affected those patients' survival prognosis. The resistance to chemoradiotherapy in rectal cancer is difficult to predict. This study was aimed at evaluating the role of V-set and transmembrane domain containing 2 like protein (VSTM2L) in resistance to chemoradiotherapy in rectal cancer.

**Methods:**

Analysis of the GEO profiling datasets of rectal cancer patients receiving pCRT disclosed that VSTM2L as a candidate gene was significantly upregulated in nonresponders of rectal cancer with pCRT. The mRNA and protein expression of VSTM2L was detected by quantitate real-time PCR, western blotting, and immunohistochemistry in six rectal cancer biopsy tissues before pCRT. Furthermore, the rectal cancer patient-derived organoids were cultured to evaluate the association of VSTM2L expression and tumor response to CRT. Overexpression of VSTM2L in cancer cells treated with CRT was analyzed for the function of cell proliferation and viability, clone formation, DNA damage repair, and apoptosis ability. The GSEA and RNA-sequence analysis were used to find the downstream mechanism of VSTM2L overexpression in cells treated with CRT.

**Results:**

The mRNA levels of VSTM2L were significantly downregulated in normal rectal tissues compared to tumor tissues and were upregulated in nonresponders of rectal cancer patients receiving pCRT and positively correlated with poor survival prognosis from GEO datasets. High expression of VSTM2L was significantly associated with tumor regression after pCRT (*P* = 0.030). Moreover, high expression of VSTM2L reduced *γ*-H2AX expression in rectal cancer patient-derived organoids treated with CRT. The overexpression of VSTM2L in colorectal cancer cells induced resistance to CRT via promoting cell proliferation and inhibiting apoptosis. The molecular mechanism revealed that the overexpression of VSTM2L induced resistance to CRT through downstream IL-4 signaling affecting the progress of cell proliferation and apoptosis.

**Conclusion:**

The high expression of VSTM2L induced resistance to CRT, and adverse survival outcomes served as a prognostic factor in patients with rectal cancer receiving pCRT, suggesting that VSTM2L high expression may be a potential resistant predictable biomarker for LARC patients receiving pCRT.

## 1. Introduction

Colorectal cancer (CRC) is viewed as the third most common cancer and the fourth cancer-related mortality in men and women worldwide [1]. And rectal cancer accounts for approximately one-third of CRC, which results in poor survival prognosis in patients. For the patients with an early stage of rectal cancer, surgical treatment is a main strategy. However, for the patients of locally advanced rectal cancer, preoperative chemoradiotherapy (pCRT) is the standard protocol for patients due to its advantages of superior survival and functional preservation in tumor invasion according to the clinical practice guideline of NCCN (National Comprehensive Cancer Network) in rectal cancer [2–5]. Nevertheless, up to 15-20% of these rectal cancer patients receiving pCRT usually appeared to have poor tumor response and regression according to the tumor regression grade (TRG) standard at the seventh edition manual of the American Joint Committee on Cancer (AJCC), which eventually led to developing local tumor recurrence or distant tumor metastasis [6, 7]. Therefore, it is important to identify potential biomarkers of locally advanced rectal cancer patients for prediction in tumor response to pCRT for further treatment of novel target therapies.

Currently, a lot of previous studies have reported that genes SERPINB5, CHD4, TCN1, VNN1, EPHA4, PCSK1, and DUOX2 as prognostic biomarkers were proven to correlate with poor tumor response and survival prognosis in patients with rectal cancer receiving pCRT [8–14]. Initially, we particularly focused on significant genes associated with poor tumor response through data mining in the Gene Expression Omnibus (GEO) database and identified potential biomarkers to predict the tumor response of treatment. Therefore, we focused on a gene V-set and transmembrane domain containing 2 like (VSTM2L) as a molecular target for further validation in the mRNA and protein level.

VSTM2L, previously known as C20orf102, is a gene of uncharacterized function to modulate neuroprotective activity and interact with humanin in both yeast and mammalian cells, which is selectively expressed in the central nervous system [15]. However, this gene's functional role and mechanism are still unclear, especially in cancer. In recent years, there are a few studies that described the expression and clinical relevance of VSTM2L in cancer; the mRNA level of VSTM2L is lower in gastric cancer tissues than in adjacent normal tissues and was downregulated in the H. pylori-positive gastric cancer [16]. Moreover, the VSTM2L gene was considered a novel CIMP-related prognostic marker to classify gastric cancer patients into high- and low-risk groups with significant difference in overall survival time [17]. In this study, we have found that high expression of VSTM2L could be significantly identified to predict poor tumor response and survival prognosis of rectal cancer patients receiving pCRT and described its important functional roles and potential mechanism of VSTM2L in rectal cancer with CRT based on the experimental evidences of patient-derived tumor organoid model and cell line.

## 2. Methods

### 2.1. Gene Expression Profiles from GEO Datasets

To identify potential genes involving in tumor response to pCRT, the transcriptomic data from microarray datasets (GSE45404, GSE68204, and GSE87211) of rectal cancer patients receiving pCRT were downloaded from the Gene Expression Omnibus (GEO) database. According to the tumor regression grade (TRG) standard at the seventh edition manual of the American Joint Committee on Cancer (AJCC), TRG1 and TRG2 are defined as response, and TRG3, TRG4, and TRG5 are defined as nonresponse or poor response. The GEO datasets were composed of 22 cases of nonresponders (NR) and 26 cases of responders (R) in GSE45404, 27 cases of nonresponders (NR) and 22 cases of responders (R) in GSE68204, and 160 cases of normal tissues and paired 203 cases of tumor tissues in GSE87211, respectively, which are shown in Table 1. We finally selected those with significance of *P* < 0.05 and alteration of log_2_(fold change) > 1 in upregulation of genes of nonresponders (NR) from those datasets for further analysis.

### 2.2. Patient Tissue Samples and Cell Culture

This study obtained the understanding and written consent of each participant and was approved by the Institutional Research Medical Ethics Committee of Nanfang Hospital. All of the participants received standard treatment of chemoradiotherapy before surgery. All samples were collected by using a colon-endoscopic biopsy from participants in the Department of General Surgery, Nanfang Hospital, Southern Medical University. These formalin-fixed paraffin-embedded (FFPE) tissue specimens were collected from rectal cancer patients receiving pCRT as verificative tissue samples. According to the TRG standard, those tissue specimens from patients were divided into two groups including response and nonresponse groups. Human colorectal cell lines HT29, HCT116, LS174T, SW480, and SW837 were purchased from American Type Culture Collection (ATCC, USA) and were cultured in RPMI1640 medium (Gibco, Carlsbad, CA) with 10% fetal bovine serum, 100 U/mL penicillin sodium, and 100 mg/mL streptomycin sulfate in humidified 5% CO_2_ at 37°C. The cells were treated with 5-FU (40 *μ*g/mL) and radiation (a single dose of 4 Gy) for 24 hours and then harvested for further experiments.

### 2.3. Quantitate Real-Time PCR and Western Blotting

Total RNA was isolated and reversed transcribed. The primer sequences are shown in Table S1. Quantitate real-time PCR were carried out according to the manufacturer's instructions. mRNA expression was normalized to the expression of GAPDH by the 2-*ΔΔ*Ct. In western blotting assays, proteins were separated on SDS/PAGE gels and transferred to PVDF membranes. The membranes were incubated with primary antibody shown in Table S1 at 4°C overnight. And then, after incubation with the secondary antibody, the membranes were visualized via the Luminate Chemiluminescent Detection Kit (Millipore, USA).

### 2.4. Immunohistochemistry

The rectal cancer tissue samples were fixed in formalin and then were embedded in paraffin. After slicing into 4 *μ*m thick sections, those sections were deparaffinized and endogenous peroxidase activity was eliminated, followed by antigen retrieval and blockade. The slices were incubated with VSTM2L primary antibodies (1 : 500, PA5-60529, Invitrogen) at 4°C overnight. And then, these slices were incubated with secondary antibody and visualized using a DAB kit.

### 2.5. Cell Transfection

An optimized VSTM2L-overexpressed lentivirus (LV-VSTM2L; GeneChem, China) was used to transfect the LS.174T and SW837 cells. The VSTM2L-overexpressing cell line was constructed using the VSTM2L-overexpressed vectors (LV-VSTM2L) and control NC (LV-NC) cell line. The efficiency of cell transfection was detected by quantitate real-time PCR in the mRNA level and western blotting in the protein level.

### 2.6. Cell Proliferation and Colony Formation Assay

The Cell Counting Kit-8 (CCK8) (Dojindo, Japan) was used to assess cell proliferation ability according to the manufacturer's instructions. A total of 1 × 10^3^ transfected cells per well were seeded into 96-well plates, and cell viability was evaluated every day following the manufacturer's protocol.

For the colony formation assay, a total of 500 transfected cells were seeded into six-well plates. After being cultured for two weeks, cell colonies were fixed with methanol and stained with 0.1% crystal violet.

### 2.7. Immunofluorescence

The cells were cultured into microscope slides overnight, and 24 hours later, slides were added 5-FU (40 *μ*g/mL) and irradiated at a single dose of 4 Gy. And then, 24 hours later, cells in slides were fixed and then permeabilized for 10 min and blocked with 5% bovine serum albumin. Slides were incubated with *γ*-H2AX primary antibody (1 : 1000, ab11174, Abcam) at 4°C overnight, followed by incubation with secondary antibody (1 : 500; Dylight 594, Abbkine). And the slides were analyzed and imaged in a laser scanning confocal microscope.

### 2.8. Cell Apoptosis Assay

The transfected LS.174T and SW837 cells were harvested after 5-FU (40 *μ*g/mL) and radiation (a single dose of 4 Gy) treatment for 24 h. Cells were collected, and apoptosis was detected by using the Annexin V-Alexa Fluor 647/PI Apoptosis Detection Kit (YESEN, Shanghai, China). The detailed procedures were performed according to the instructions provided.

### 2.9. Tumor Organoid Culture

Human rectal cancer patient-derived tumor organoids were cultured as described in a previous study [18, 19]. Fresh rectal cancer tissue was washed with PBS and then resected into 1 mm pieces in PBS-DTT buffer. The pieces were digested in mixed medium that consisted of advanced DMEM/F12 with 2% FBS, Pen/Strep, 100 U/mL collagenase type XI, and 125 *μ*g/mL dispose type II at 37°C for 40 min and then added TrypLE Express and DNase I medium for further digestion for 10 min. These samples were then embedded in Matrigel and cultured, filtered through a 70 *μ*m cell strainer, and centrifuged at 300 × g for 5 min, and the isolated tumor cells were embedded in Matrigel, depending on the pellet size. After being cultured for >2 weeks with high viability, these tumor organoids were treated with 5-FU (40 *μ*g/mL) and radiation (a single dose of 4 Gy) for 48 hours.

### 2.10. Statistical Analysis

SPSS 22.0 for Windows was used for statistical analysis. Kaplan-Meier curves were plotted to evaluate the impact of gene expression on survival time (OS) and disease-free survival (DFS) by GraphPad Prism 8. *P* value < 0.05 was considered to have statistical significance.

## 3. Results

### 3.1. High Expression of VSTM2L Correlated with Poor Response and Prognosis in Rectal Cancer Patients Receiving pCRT

**Figure 1:**
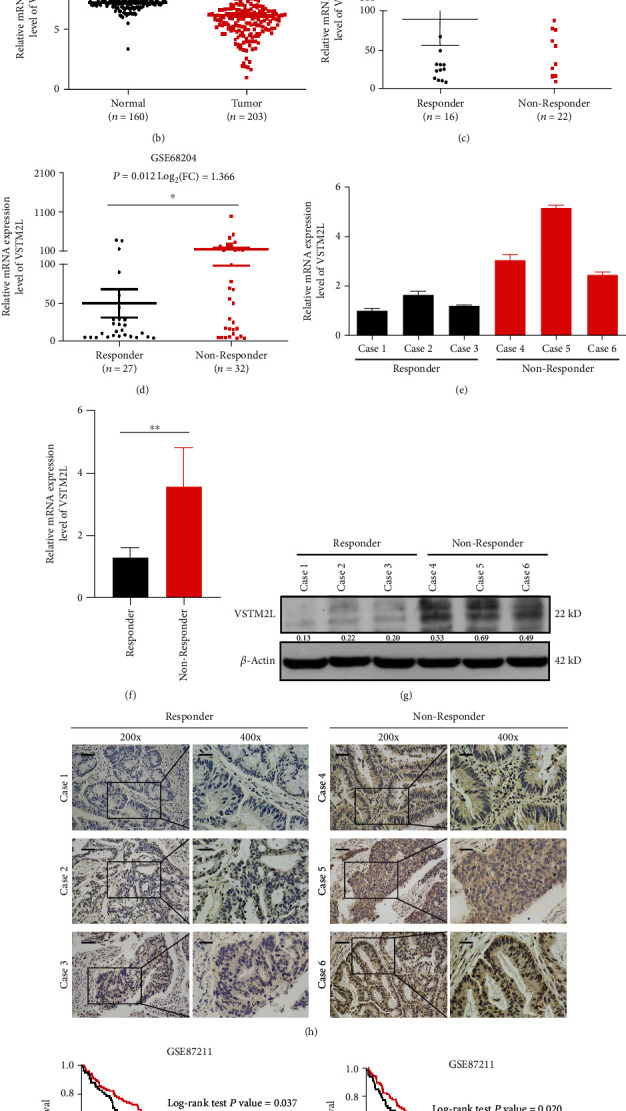
High expression of VSTM2L correlates with poor tumor response and prognosis in patients with rectal cancer receiving pCRT. (a) Venn diagram analysis of GEO datasets GSE45404, GSE68204, and GSE87211 about rectal cancer patients treated with pCRT showed 9 upregulated genes in nonresponders to pCRT between response (R) and nonresponse (NR) groups in GSE45404 and GSE68204 and significantly different genes between normal (N) and tumor (T) groups in GSE87211. The standard of selection is on the significance of *P* < 0.05 and alteration of log_2_(fold change) > 1. (b) The mRNA expression level of VSTM2L is significantly downregulated in tumor compared to normal tissues in GSE87211 (*P* = 1.5*E* − 10, log_2_FC = 1.044). (c, d) The mRNA expression level of VSTM2L is significantly upregulated in tumor tissues of the nonresponse group compared to that of the response group in GSE45404 (*P* = 0.035, log_2_FC = 1.192) and GSE68204 (*P* = 0.012, log_2_FC = 1.366), respectively. (e–h) The mRNA and protein expression level of VSTM2L in the response and nonresponse groups from 6 rectal cancer biopsy tissues. Scale bar: 200x: 50 *μ*m, 400x: 20 *μ*m. (i, j) The Kaplan-Meier survival curves of patients in GSE87211 demonstrate the significant prognostic impact of VSTM2L expression on survival time (*P* = 0.037) and disease-free time (*P* = 0.020). ^∗^*P* < 0.05, ^∗∗^*P* < 0.01, and ^∗∗∗^*P* < 0.001.

Data mining from the public transcriptome dataset of a rectal cancer patient receiving pCRT (GSE87211, GSE68204, and GSE45404) from GEO datasets, we aimed to screen differentially upexpressed genes in nonresponders between rectal cancer patients receiving pCRT for predicting the tumor response according to a standard of *P* < 0.05 and log_2_(fold change) > 1. We have found that there were nine genes significantly identified in upregulation of nonresponders to pCRT, including C6orf15, KRT23, COL2A1, FOLR1, FREM1, DACT2, NKD2, VSTM2L, and ZSCAN18 (Figure 1(a)). Among these candidate genes, VSTM2L gene was chosen for further investigation because its mRNA expression is significantly relative to both the survival time (OS) and disease-free time (DFS) in the GSE87211 dataset about rectal cancer patients receiving pCRT (Supplementary Figure (available here)). VSTM2L was significantly downregulated in tumor compared to normal tissues in GSE87211 (Figure 1(b)) and upregulated significantly in tumor tissues of nonresponders to pCRT in the GSE45404 and GSE68204 datasets (Figures 1(c) and 1(d)). To identify and validate the relationship between VSTM2L expression and tumor response of rectal cancer patients receiving pCRT, we have found that high expression of VSTM2L is positively correlated with poor tumor response through mRNA and protein level detection of six rectal cancer patients receiving pCRT (Figures 1(e)–1(h)). The survival analysis revealed that high expression of VSTM2L is associated with poor prognosis in rectal cancer patients receiving pCRT (Figures 1(i) and 1(j)). In addition, the analysis of association between clinical characteristics and VSTM2L expression in 186 rectal cancer patients in GSE87211 showed that high expression of VSTM2L was significantly associated with tumor regression after pCRT (*P* = 0.03) (Table 2). The high expression of VSTM2L may have potential to predict poor tumor response and prognosis in rectal cancer patients receiving pCRT.

### 3.2. High Expression of VSTM2L Reduced *γ*-H_2_AX Expression in Rectal Cancer Patient-Derived Organoids Treated with CRT

**Figure 2:**
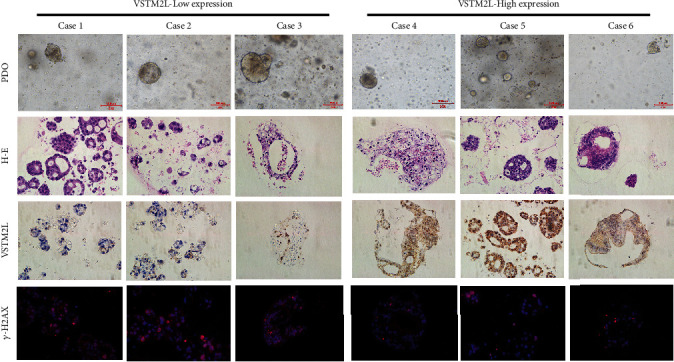
The association between VSTM2L expression and tumor response with CRT through the detection of *γ*-H2AX expression in rectal cancer patient-derived organoids cultured and treated with CRT.

To further explore the association of VSTM2L expression and tumor response to CRT, we preoperatively collected fresh tumor tissues from rectal cancer patients receiving pCRT to culture for patient-derived organoid in vitro. The expression of VSTM2L and DNA damage marker *γ*-H_2_AX was detected in these patient-derived organoids, and then, they were treated with CRT. The results revealed that the patient-derived organoids with high expression of VSTM2L significantly reduced *γ*-H_2_AX expression compared to the organoids with low expression of VSTM2L when these organoid models were treated with CRT (Figure 2). High expression of VSTM2L may induce resistance to CRT when the rectal cancer patient-derived organoid was treated with CRT.

### 3.3. Overexpression of VSTM2L Induced Resistance to CRT in Cancer Cells via Promoting Cell Proliferation and Inhibiting Cell Apoptosis

**Figure 3:**
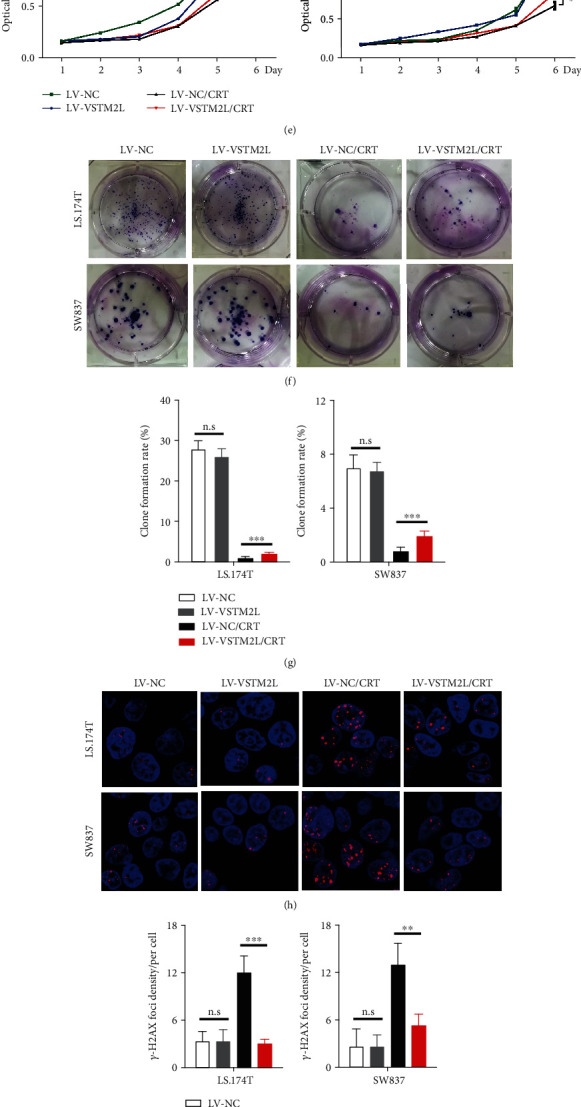
Overexpression of VSTM2L induced resistance to CRT in colorectal cancer cells in vitro. (a, b) The endogenous mRNA and protein levels of VSTM2L expression in 5 colorectal cancer cell lines. (c, d) The transfected effects of VSTM2L overexpression in LS.174T and SW837 cells were analyzed by qRT-PCR and western blot analysis. GAPDH was used as the loading control. ^∗∗^*P* < 0.01 and ^∗∗∗^*P* < 0.001. (e) The effect of overexpression of VSTM2L in LS.174T and SW837 cells treated with CRT, as determined by the CCK-8 assay. The results are shown as the means ± SEMs (*n* = 3), ^∗^*P* < 0.05, ^ns^*P* > 0.05. (f, g) The effect of overexpression of VSTM2L in CRC cells treated with CRT on colony formation assays. The results are shown as means ± SEMs (*n* = 3), ^∗∗∗^*P* < 0.001, and ^ns^*P* > 0.05. (f, g) The effect of overexpression of VSTM2L in CRC cells treated with CRT on *γ*-H_2_AX expression to evaluate the ability of DNA damage. The results are shown as means ± SEMs (*n* = 3), ^∗∗^*P* < 0.01, ^∗∗∗^*P* < 0.001, and ^ns^*P* > 0.05. (j, k) VSTM2L overexpression in CRC cells treated with CRT significantly inhibited cell apoptosis, as determined by Annexin V-APC/PI staining and flow cytometry. ^∗∗∗^*P* < 0.001 and ^ns^*P* > 0.05.

Given previous results, we further investigate the functional role of VSTM2L high expression in cancer cells with chemoradiation treatment (CRT); we analyzed the endogenous mRNA and protein levels of VSTM2L expression in five colorectal cancer cell lines by quantitative real-time PCR and western blotting (Figures 3(a) and 3(b)). The results showed that the LS.174T and SW837 cells with relatively low endogenous VSTM2L expression were selected to establish stable VSTM2L-overexpressing cell lines (Figures 3(c) and 3(d)). As proliferation was evidenced by CCK-8 assays and colony formation assays, overexpression of VSTM2L in LS.174T and SW837 cells treated with CRT in vitro significantly promoted cell proliferation and viability, while not significantly promoting cell growth and proliferation without CRT (Figures 3(e)–3(g)). Moreover, *γ*-H_2_AX assays revealed that overexpression of VSTM2L in LS.174T and SW837 cells treated with CRT significantly reduced *γ*-H_2_AX expression compared to control cells but did not significantly inhibit *γ*-H_2_AX expression without CRT (Figures 3(h) and 3(i)). Furthermore, we analyzed cell apoptosis by flow cytometry which revealed that overexpression of VSTM2L suppressed a significant increase in the total cell apoptosis rate in the LS.174T and SW837 cells treated with CRT. However, overexpression of VSTM2L in the LS.174T and SW837 cells has not significantly increased the total cell apoptosis rate when those cells were treated without CRT (Figures 3(j) and 3(k)). Taken together, these results indicated that overexpression of VSTM2L induced resistance to CRT in colorectal cancer cells through promoting cell proliferation and DNA damage repair and inhibiting cell apoptosis when cancer cells were treated with CRT.

### 3.4. Overexpression of VSTM2L Induced Resistance to CRT through Downstream IL-4 Signaling

**Figure 4:**
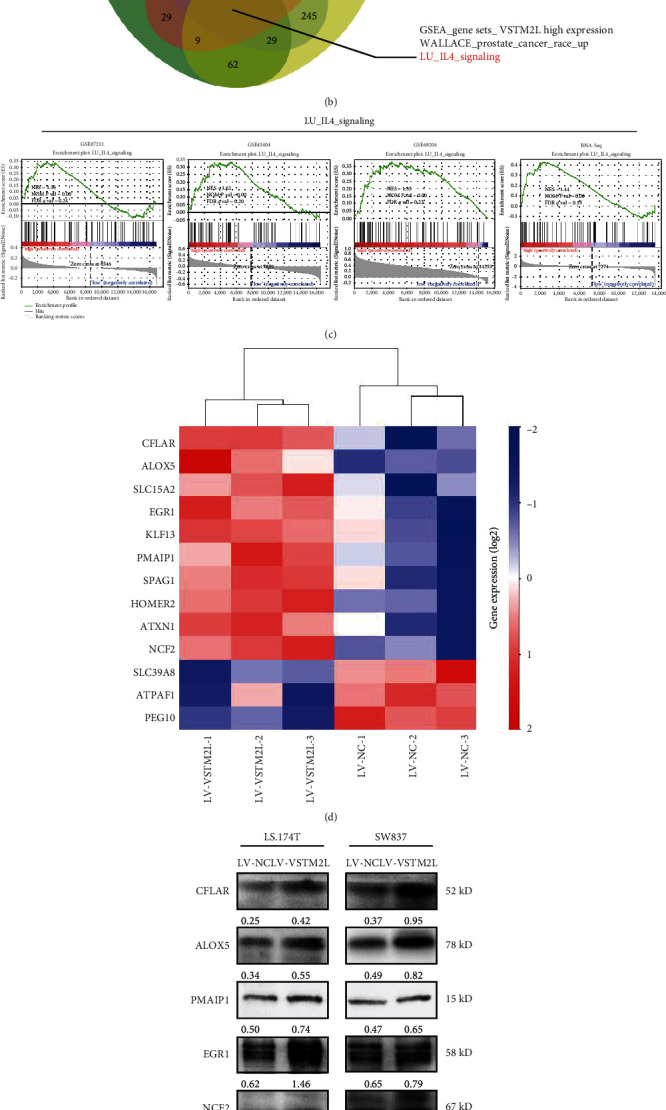
Overexpression of VSTM2L induced resistance to CRT through downstream IL-4 signaling. (a) The classification of rectal cancer patients and LS.174 cancer cell was divided into high and low expression groups from GSE45404, GSE68204, GSE87211, and VSTM2L-overpressing cell RNA-sequence data according to the mRNA expression level of VSTM2L. ^∗∗∗^*P* < 0.001. (b, c) Upregulation of VSTM2L expression was positively correlated with IL-4 signaling in rectal cancer as predicted by GSEA (GSEA: gene set enrichment analysis; NES: normalized enrichment score > 1, *P* value < 0.001). (d) The heat map showed that the overexpression of VSTM2L was associated with downstream IL-4 signaling enrichment-related 13 candidate genes as the RNA-Seq data analysis (*P* < 0.05), including 10 upregulated genes and 3 downregulated genes. (e) Overexpression of VSTM2L in LS.174T and SW837 cell affected the protein expression of downstream IL-4 signaling genes which proved the effects of promoting cell proliferation and inhibiting apoptosis.

To explore the underlying downstream molecular mechanisms that VSTM2L overexpression induces resistance to CRT in cancer cells, we performed gene set enrichment analysis (GSEA) on the microarray data from GSE45404, GSE68204, GSE87211, and VSTM2L-overexpressing RNA sequence. These datasets were classified into two groups according to the mRNA expression level of VSTM2L (Figure 4(a)). The GSEA results showed that high expression of VSTM2L was mostly positively associated with genes involved in IL-4 signaling (Figures 4(b) and 4(c)). We then used our own VSTM2L-overexpressing RNA-Seq data to perform the pathway relative genes of IL-4 signaling and found that the mRNA level of 13 genes has appeared to significantly change (*P* < 0.05), including 10 upregulated genes including CFLAR, ALOX5, SLC15A2, EGR1, KLF13, PMAIP1, SPAG1, HOMER2, ATXN1, and NCF2 and 3 downregulated genes including SLC39A8, ATPAF1, and PEG10 (Figure 4(d)). Among these significant genes of IL-4 signaling are CFLAR, ALOX5, PMAIP1, EGR1, NCF2, SLC39A8, and PEG10 which have been reported to be associated with tumor progression or chemotherapy-drug resistance through effecting proliferation and apoptosis in previous studies. And then, we further verified the impact of VSTM2L overexpression in those reporting proliferation and apoptosis relative genes of IL-4 signaling in LS.174T and SW837 cells; the results suggested that VSTM2L overexpression significantly upregulated the protein expression of CFLAR, ALOX5, PMAIP1, EGR1, and NCF2 and downregulated the protein expression of SLC39A8 and PEG10 (Figure 4(e)). Thus, we have identified that overexpression of VSTM2L induced resistance to CRT in cancer cells through downstream IL-4 signaling which could affect the progress of cell proliferation and apoptosis.

## 4. Discussion

Preoperative chemoradiotherapy (pCRT) has been the mainstay treatment for locally advanced rectal cancer patients, but the therapeutic effects have differed widely among persons and affect patient's survival prognosis. Therefore, identification of predictive and prognostic markers for the preoperative chemoradiotherapy treatment options is needed. Many studies have focused on identifying genes as biomarkers associated with tumor response and survival prognosis of rectal cancer patients with pCRT, such as SERPINB5, CHD4, TCN1, VNN1, EPHA4, PCSK1, and DUOX2 genes [8–14]. Those studies have only showed the relationship between gene expression and tumor response or prognosis but have not indicated the functional role and molecular mechanism of candidate gene in rectal cancer treated with CRT. The aim of this study was to find resistant-related genes and indicate functional role and potential mechanism in rectal cancer with CRT. Eventually, VSTM2L was chosen as a candidate gene for further investigation after GEO dataset analysis.

VSTM2L is a novel molecule described as an uncharacterized function gene in cancer. Although a few studies have been reported about its gene expression and clinical relevance in gastric cancer [16, 17], there is still lack of researches on the functional role and potential molecular mechanism of VSTM2L in cancer. In our study, we used GEO dataset analysis and considered VSTM2L high expression as a biomarker for prediction of poor tumor response and survival prognosis of rectal cancer patients receiving pCRT. We found that VSTM2L high expression is positively associated with poor tumor response and survival prognosis in rectal cancer patients receiving pCRT. In addition, we cultured rectal cancer patient-derived tumor organoid to identify the association of VSTM2L expression and tumor response when those tumor organoids were treated with CRT in vitro. And then, we constructed VSTM2L-overexpressing colorectal cancer cells in vitro to investigate the functional role of VSTM2L high expression. The overexpression of VSTM2L promoted cell proliferation and inhibited cell apoptosis when treated with CRT. The overexpression of VSTM2L induced resistance to CRT in cancer cells. The molecular mechanism revealed that overexpression of VSTM2L is significantly positively associated with IL-4 signaling with GSEA and affects the expression of downstream IL-4 signaling genes CFLAR, ALOX5, PMAIP1, EGR1, NCF2, SLC39A8, and PEG10.

The overexpression of VSTM2L significantly upregulated CFLAR, ALOX5, PMAIP1, EGR1, and NCF2 protein expression and downregulated SLC39A8 and PEG10 protein expression. In previous studies, IL-4 and IL-4 signaling genes play crucial roles and associate with the progression of colorectal cancer. IL-4 induced STAT6 signaling in promoting cell proliferation/growth and cell apoptosis resistance and induced epithelial-mesenchymal transition (EMT) in colorectal cancer cells via E2F1/SP3/STAT6 axis [20–22]. IL-4 autocrine response mediated colorectal cancer stem-like cell (CSC) survival and chemotherapeutic resistance through protecting the tumorigenic CD133+ CSC apoptosis; blocking or inhibiting IL-4 signaling sensitizes CSCs to apoptosis and increases the efficacy of cytotoxic therapy in vivo [23, 24]. CFLAR, as an antiapoptotic protein, is a key gene regulator to inhibit TRAIL-induced apoptosis in colorectal cancer cells [25–27]. Targeting ALOX5 promotes colorectal cancer cell proliferation, and silencing of ALOX5 inhibited colorectal cancer cell growth [28, 29]. PMAIP1 is involved in the intrinsic apoptosis pathway, selectively binds to MCL1, and prevents MCL1 to inhibiting apoptosis in colorectal cancer [30]. EGR1 high expression correlates with resistance to anti-EGFR treatment in metastatic colorectal cancer patients treated with cetuximab, and silencing EGR1 expression promotes killing HCT116 colorectal cancer cells and delaying tumor growth [31, 32]. EGR1 may play an oncogenic role in colorectal cancer. There was a high level of mRNA expression of NCF2 in patients with colorectal adenocarcinoma compared with normal tissue. Loss of NCF2 increased apoptosis and generation. NCF2 is a novel regulator in the antiapoptotic functions of p53 [33, 34]. The role of SLC39A8 is the regulation of cisplatin sensitivity through antiapoptotic protein Bcl-2; loss of SLC39A8 elevated Bcl-2 expression and modulates cisplatin-induced cell apoptosis, through increasing cleaved Caspase-3 protein [35]. PEG10 is one of the upregulated genes in the tissues of rectal adenocarcinoma with metastasis when compared to corresponding tumor tissues without metastasis [36]. PEG10 overexpression promoted HCT116 colorectal cancer cell proliferation and inhibited apoptosis via increasing Wnt1 and *β*-catenin expression [37]. As the described evidences in previous reported studies, overexpression of VSTM2L in cells induced resistance to CRT through downstream IL-4 signaling and pathway-related genes such as CFLAR, ALOX5, PMAIP1, EGR1, NCF2, SLC39A8, and PEG10 via affecting the process of cell proliferation and apoptosis.

As we all know, there are no reported studies on the functional role and mechanism of VSTM2L gene in cancer; we have originally reported the VSTM2L gene function and molecular mechanism in rectal cancer with CRT according to the experimental evidence of rectal cancer patient-derived organoid and cancer cell in this study. But there is also a limitation; the correlation of VSTM2L overexpression and downstream IL-4 signaling which affect the process of cell proliferation and apoptosis is needed to further investigate based on more experiments.

In conclusion, we demonstrated that the high expression of VSTM2L was positively correlated with poor response to pCRT and prognosis in rectal cancer patients. Furthermore, the rectal cancer patient-derived tumor organoids were used to identify the association of VSTM2L expression and tumor response to CRT. Most importantly, the functional role and molecular mechanism of VSTM2L overexpression in rectal cancer with pCRT were described in this study. VSTM2L was a promising and predictive prognostic biomarker in rectal cancer. VSTM2L may act as a potential target for personalized therapy in patients with rectal cancer treated with pCRT.

## Figures and Tables

**Table 1:** GEO datasets about the preoperative chemoradiotherapy (pCRT) of rectal cancer.

Datasets	Platform	Treatment	Cases	Patients
GSE87211	GPL13497	pCRT	363	Normal (*n* = 160, 44.1%)
				Tumor (*n* = 203, 55.9%)
GSE45404	GPL4133	pCRT	38	Response (*n* = 16, 42.1%)
				Nonresponse (*n* = 22, 57.9%)
GSE68204	GPL6480	pCRT	59	Response (*n* = 27, 45.8%)
				Nonresponse (*n* = 32, 54.2%)

**Table 2:** Correlation between clinical characteristics and expression of VSTM2L in GSE87211 about preoperative chemoradiotherapy of rectal cancer (total cases = 186).

Characteristics		VSTM2L expression (%)		*P* value
		High (*n* = 73)	Low (*n* = 113)	
Age	>60	40	74	0.144
	<60	33	39	

Gender	Male	50	79	0.838
	Female	23	34	

Kras mutation	Wild type	44	61	0.398
	Mutation type	29	52	

Preoperative treatment	5-FU+RT	38	61	0.233
	5-FU+oxaliplatin+RT	35	48	
	FU+oxaliplatin+cetuximab+RT	0	4	

Invasion depth before pCRT	T2	1	4	0.166
	T3	65	105	
	T4	7	4	

Invasion depth after pCRT	T0	10	22	0.842
	T1	7	13	
	T2	18	24	
	T3	34	48	
	T4	4	6	

LN metastasis before pCRT	N0	29	33	0.137
	N1	44	80	

LN metastasis after pCRT	N0	47	86	0.217
	N1	20	20	
	N2	6	7	

Metastasis before pCRT	M0	68	108	0.474
	M1	5	5	

Metastasis after pCRT	M0	66	107	0.264
	M1	7	6	

ypTNM stage before pCRT	I+II	29	31	0.080
	III+IV	44	82	

TNM stage after pCRT	I+II	46	82	0.170
	III+IV	27	31	

Tumor downstage after pCRT	No	33	37	0.087
	Yes	40	76	

Tumor regression after pCRT	No	32	32	0.030^∗^
	Yes	41	81	

Recurrence after surgery	No	51	89	0.170
	Yes	22	24	

^∗^ *P* < 0.05 was considered significant.

## Data Availability

GEO datasets are available in PubMed (https://www.ncbi.nlm.nih.gov/gds/).

## Supplementary Materials

Supplementary 1 Supplementary Figure legend: VSTM2L was chosen as a significantly prognostic biomarker in patients with rectal cancer receiving pCRT from 9 candidate genes. Kaplan-Meier survival curves of patients in GSE87211 demonstrate the prognostic impact of significantly upregulated gene expression, including C6orf15, KRT23, COL2A1, FOLR1, FREM1, DACT2, NKD2, and ZSCAN18 on survival time and disease-free time except for the VSTM2L gene.

Supplementary 2 Supplementary Table S1: information on primer sequences and primary antibody.
