# Supplementary material for: High Expression of VSTM2L Induced Resistance to Chemoradiotherapy in Rectal Cancer through Downstream IL-4 Signaling
Source: J Immunol Res. 2021 Jan 8;2021:6657012. doi: 10.1155/2021/6657012 (PMC7811563; doi:10.1155/2021/6657012)
Supplement: Supplementary 1 — Supplementary Figure legend: VSTM2L was chosen as a significantly prognostic biomarker in patients with rectal cancer receiving pCRT from 9 candidate genes. Kaplan-Meier survival curves of patients in GSE87211 demonstrate the prognostic impact of significantly upregulated gene expression, including C6orf15, KRT23, COL2A1, FOLR1, FREM1, DACT2, NKD2, and ZSCAN18 on survival time and disease-free time except for the VSTM2L gene. [file 6657012.f1.pdf]

Supplementary. Figure.

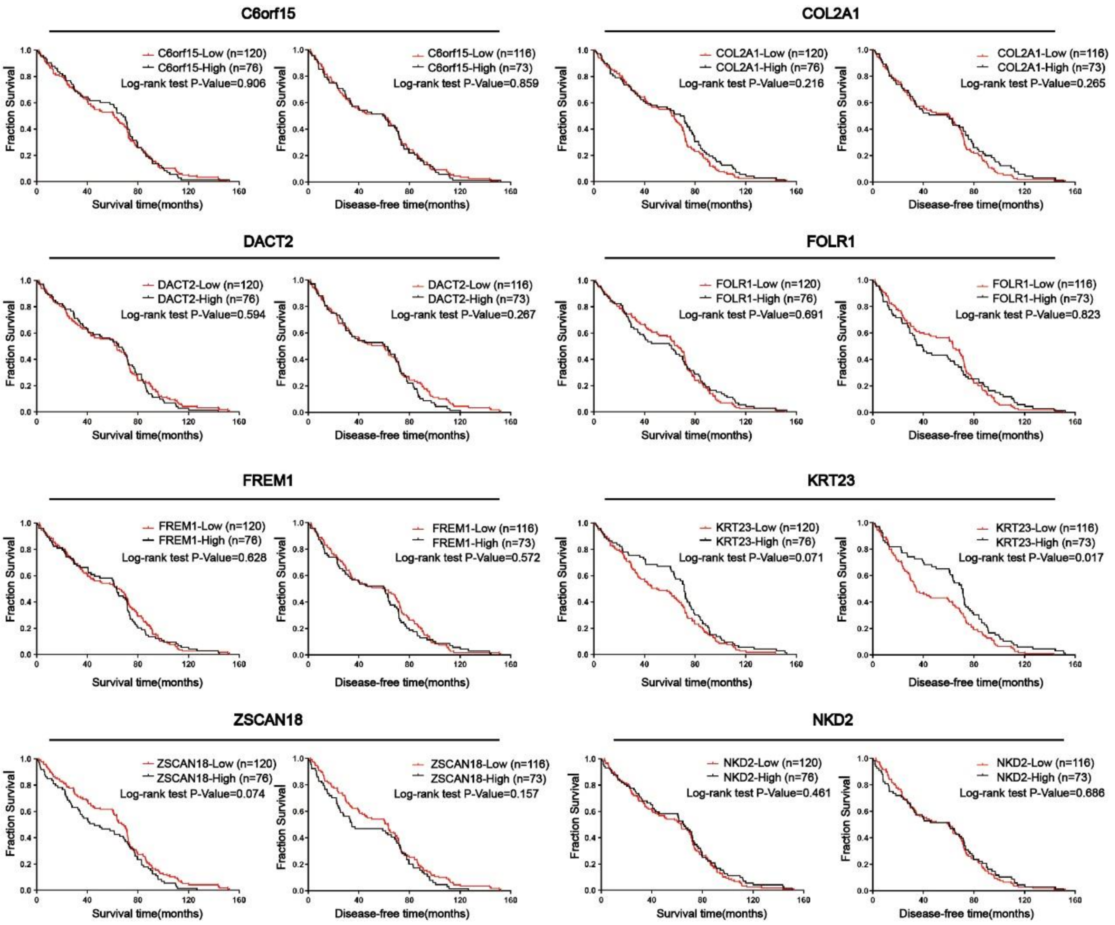

Supplementary Figure Legend

VSTM2L was chosen as a significantly prognostic biomarker in patients with rectal cancer receiving pCRT from 9 candidate genes. Kaplan-Meier survival curves of patients in GSE87211 demonstrate the prognostic impact of up-regulated significantly gene expression, including C6orf15, KRT23, COL2A1, FOLR1, FREM1, DACT2, NKD2, ZSCAN18 on survival time and disease-free time except for VSTM2L gene.

Supplymetary.Table.S1 Information of primer sequences and primary antibody

Primer Sequences for Quantitate real-time PCR	
VSTM2L-Forward	5'-GATCCAGTGGTGGTATGTACGG-3'
VSTM2L-Reverse	5'-ACACTTATTTTGGTTGCCTCCTTC-3'
GAPDH--Forward	5'-ATTCCACCCATGGCAAATTCC-3'
GAPDH--Reverse	5'-GACTCCACGACGTACTCAGC -3'
Primary Antibody for Western-Blotting	
VSTM2L	1:500, 25457-1-AP, Proteintech Group
CFLAR	1:1000,10394-1-AP, Proteintech Group
ALOX5	1:500, 10021-1-Ig, Proteintech Group
PMAIP1	1:1000, NB600-1159, Novus Biologicals
EGR1	1:1000, 55117-1-AP, Proteintech Group
NCF2	1:500, 15551-1-AP, Proteintech Group
SLC39A8	1:1000, 20459-1-AP, Proteintech Group
PEG10	1:1000, 14412-1-AP, Proteintech Group
GAPDH	1:5000, 60004-1- Ig, Proteintech Group
