# Supplementary material for: High Expression of VSTM2L Induced Resistance to Chemoradiotherapy in Rectal Cancer through Downstream IL-4 Signaling
Source: J Immunol Res. 2021 Jan 8;2021:6657012. doi: 10.1155/2021/6657012 (PMC7811563; doi:10.1155/2021/6657012)
Supplement: Supplementary 2 — Supplementary Table S1: information on primer sequences and primary antibody. [file 6657012.f2.docx]

| Primer Sequences for Quantitate real-time PCR | |
| --- | --- |
| VSTM2L-Forward | 5'-GATCCAGTGGTGGTATGTACGG-3' |
| VSTM2L-Reverse | 5'-ACACTTATTTTGGTTGCCTCCTTC-3' |
| GAPDH--Forward | 5'-ATTCCACCCATGGCAAATTCC-3' |
| GAPDH--Reverse | 5'-GACTCCACGACGTACTCAGC -3' |
| Primary Antibody for Western-Blotting | |
| VSTM2L | 1:500, 25457-1-AP, Proteintech Group |
| CFLAR | 1:1000,10394-1-AP, Proteintech Group |
| ALOX5 | 1:500, 10021-1-Ig, Proteintech Group |
| PMAIP1 | 1:1000, NB600-1159, Novus Biologicals |
| EGR1 | 1:1000, 55117-1-AP, Proteintech Group |
| NCF2 | 1:500, 15551-1-AP, Proteintech Group |
| SLC39A8 | 1:1000, 20459-1-AP, Proteintech Group |
| PEG10 | 1:1000, 14412-1-AP, Proteintech Group |
| GAPDH | 1:5000, 60004-1- Ig, Proteintech Group |

**Supplymetary.Table.S1** Information of primer sequences for QPCR assay and primary antibody for Western-Blot assay.
